# Reducing Porosity and Refining Grains for Arc Additive Manufacturing Aluminum Alloy by Adjusting Arc Pulse Frequency and Current

**DOI:** 10.3390/ma11081344

**Published:** 2018-08-03

**Authors:** Donghai Wang, Jiping Lu, Shuiyuan Tang, Lu Yu, Hongli Fan, Lei Ji, Changmeng Liu

**Affiliations:** School of Mechanical Engineering, Beijing Institute of Technology, Beijing 100081, China; wdh4043@163.com (D.W.); jipinglu@bit.edu.cn (J.L.); yulu2219@163.com (L.Y.); fanhongli2000@sina.com (H.F.); jl12199@163.com (L.J.); liuchangmeng@bit.edu.cn (C.L.)

**Keywords:** arc additive manufacturing, Al–5Si alloy, pulse frequency, arc current, microstructure, porosity

## Abstract

Coarse grains and gas pores are two main problems that limit the application of additive manufacturing aluminum alloys. To reduce porosity and refine grains, this paper presents a quantitative investigation into the effect of pulse frequency and arc current on the porosity and grains of arc additive manufacturing Al–5Si alloy. The experiment results show that pulse frequency and arc current have a significant impact on the macrostructure, microstructure, porosity, and tensile properties of the samples. Fine grains and a uniform microstructure can be obtained with low pulse frequency and low arc current as a result of the rapid cooling of the molten pool. With the increase of pulse frequency, density shows a trend that firstly escalates and attains the maximum value at 50 Hz, but later declines as a result of the relation between pores formation and gas escape. Moreover, better tensile properties can be obtained at low pulse frequency and low arc current because of the finer grains.

## 1. Introduction

Over the last few decades, researchers have already made great strides in metal additive manufacturing technologies [1,2,3], which can be classified by the employed heat source, including laser additive manufacturing, electron beam additive manufacturing, and arc additive manufacturing. Williams et al. [4] pointed out that, compared with other additive manufacturing methods, arc additive manufacturing has distinct advantages: High manufacturing efficiency, low cost (machine and materials), and good structural integrity. The arc additive manufacturing process often employs wire as feedstock, compared with other powder-feed additive manufacturing process, it can produce large scale metallic parts with a much higher deposition rate [5]. Consequently, the research of arc additive manufacturing has become a hotspot. Arc additive manufacturing has been widely used to fabricate titanium alloy, stainless steel, and nickel alloy [6,7]. For aluminum alloys, which are widely used in the aerospace and automobile industry owing to their excellent performance, such as high strength-to-weight ratio, high formability, and high durability [8,9,10], their applications of additive manufacturing are still limited. Some recent studies have been devoted to the process parameters on the quality and properties of arc additive manufacturing aluminum alloy [11,12]. However, the main reason that limited the aluminum’s application of additive manufacturing is that gas pores and coarse grains easily form during arc additive manufacturing, which largely reduce the mechanical properties of aluminum alloys [13,14,15,16]. Some researchers employed different cold metal transfer processes and pure argon flow rates in order to reduce the pores and obtain refine microstructure in arc additive manufacturing of Al–Cu alloy [17,18].

Among the alumina alloys, Al–Si alloys have been widely used in automotive and aerospace industries because of their excellent characteristics such as excellent cast ability and mechanical properties [19]. However, studies focused on the reducing porosity and improving properties of Al–Si alloys have rarely been reported. Thus, the investigation on reducing the gas pores and refining grains is meaningful, which allows a broader application of Al–Si alloys.

The formation of gas pores and coarse grains are intensely associated with the melting pool, which is influenced by the arc pulse frequency and arc current [20]. Hence, in this study, the effect of pulse frequency and arc current on the gas pores and grains has been systematically investigated. The mechanism of reducing porosity and refining grains was revealed by adjusting the pulse frequency and arc current. Meanwhile, the geometry, microstructures, and tensile properties under different pulse frequencies and arc currents will be investigated.

## 2. Experimental Details

**Figure 1:**
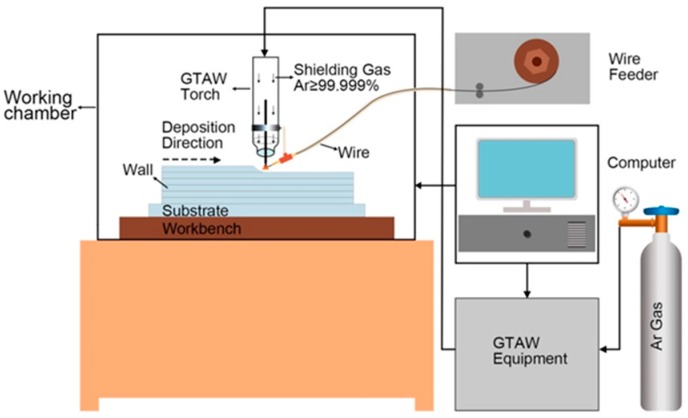
Experimental set-up for arc additive manufacturing.

Figure 1 schematically expressed the experimental setup for arc additive manufacturing. The system consists of a gas tungsten arc welding (GTAW) equipment (Miller Dynasty 350, Miller Electric Manufacturing Co., Appleton, WI, USA), a wire feeder (Jetline 9700 W, Miller Electric Manufacturing Co., Appleton, WI, USA), a computer, a three-dimensional workbench, and a working chamber.

Al–5Si alloy wire with a diameter of 1.2 mm was employed in this work (ER4043, chemical composition is shown in Table 1). The rolled pure aluminum substrate was mechanically cleaned and fixed on the workbench before the deposition process. Based on careful analysis of previous experiments about the GTAW of Al–5Si alloy, optimized fabrication parameters were determined (Table 2), then ten thin walls with 15 layers were fabricated, which were built layer by layer.

**Figure 2:**
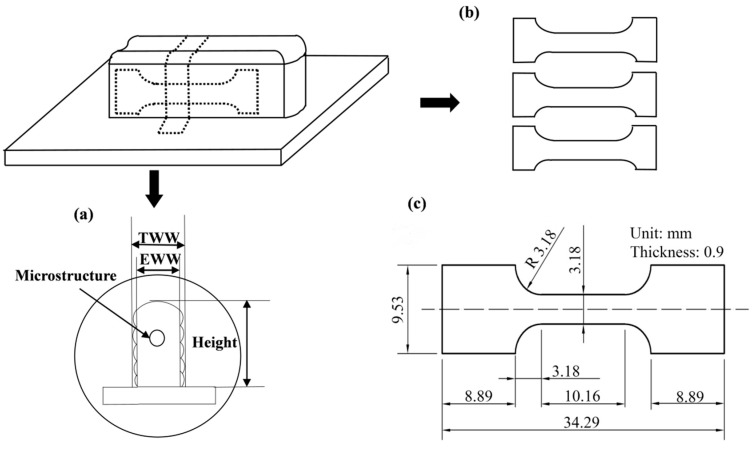
Test samples: (**a**) Sketch of layer thickness and width; (**b**) tensile specimens; (**c**) dimensional sketch of tensile specimens. TWW—total wall width; EWW—effective wall width.

The following analyses are performed to investigate the specimens manufactured under different pulse frequencies and currents in terms of macrostructure, microstructure, porosity, and tensile properties. The sketch of layer thickness and width is shown in Figure 2a. Generally speaking, there are two different definitions of the width of deposition alloy. TWW refers to the total wall width and EWW refers to effective wall width as shown in Figure 2a. In this study, TWW is selected as the width of deposited wall, layer thickness is referred to the average of 15 layers. For microstructural observations, test samples were taken from the middle of deposited walls, inlayed, and ground with silicon carbide papers, then the samples were polished by electro-polishing. After that, samples were etched using a modified Keller’s reagent (except the specimens used for the porosity characterization), and observed using an optical microscope (OM, Leica DM4000M, Leica Microsystems Inc., Buffalo Grove, IL, USA). Then, the dendrite arm spacing, and the numbers and cross-section areas of gas pores were measured by 10 OM images with a magnification of 100 from five cross-sections for each deposited wall, and the statistical data were obtained by Image Pro Plus software (6.0, Media Cybernetics, Warrendale, PA, USA). Here, density is represented by the ratio of total area of gas pores to total area of 10 OM images. For tensile properties, three tested tensile specimens (parallel to the longitudinal direction of the deposited walls) were selected from each deposited wall and then tested by tensile test machine (Instron5966, Instron, Norwood, CO, USA) at room temperature (the cross-head speed was 0.01 mm/s, and a dynamic strain gauge extensometer was applied to record the strain). The average tensile data was adopted and the fractured samples were observed by scanning electron microscope (SEM, JSM-6610LV, JEOL, Tokyo, Japan). The dimensions of tensile specimens are shown in Figure 2c.

## 3. Results and Discussion

### 3.1. Effects of Pulse Frequency

#### 3.1.1. Macrostructure

**Figure 3:**
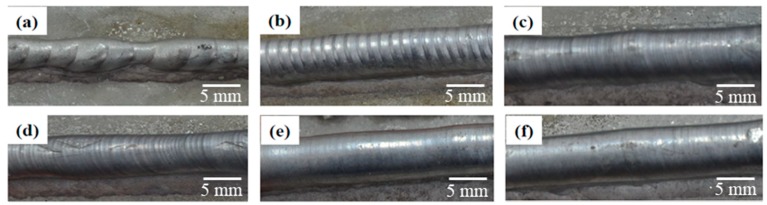
Surface morphology of the walls deposited by (**a**) 2 Hz; (**b**) 5 Hz; (**c**) 10 Hz; (**d**) 50 Hz; (**e**) 200 Hz; and (**f**) 500 Hz.

From Figure 3, it is found that the geometry and surface morphology of the deposited walls change significantly with the alteration of pulse frequency. Much more coarse periodic ripples are observed at the surface under low pulse frequency, as shown in Figure 3a,b. These coarse periodic ripples are indicative of the typical patterns of interfaces caused by solidified droplet. Besides, fine striations are observed on the surface of the deposited walls at high pulse frequency, as shown in Figure 3c–f. Only very few surface ripples with a low amplitude are found on the surface of the deposited part under the high pulse frequency. The effect of pulse frequency on the surface of deposited walls can be understood as follows: During the manufacturing process, Al–5Si alloy melted at peak current period and droplet will drip on the substrate or deposited wall as a result of the surface tension during the background current. Hence, it takes more time to become bigger at low pulse frequency, so the droplets are usually larger and the number of the droplets is fewer than that at high pulse frequency. This results in coarse periodic ripples on the surface.

Table 3 lists the effect of the pulse frequency on the layer thickness and width of the deposited walls. In general, with the pulse frequency increasing from 2 Hz to 500 Hz, the layer thickness decreases and the width increases. As known, the variation of geometry mainly depends on the size of the molten pool that is strongly affected by heat input and heat loss [21]. In this study, under different pulse frequencies, the total value of heat input is kept constant while the conditions of heat loss are different. During the peak current period, high heat input results in the formation of the melting pool [22]. During the background current period, the melting pool cools down rapidly with lots of heat loss. For low pulse frequency, the background current time during a single pulse is long, which is beneficial for heat loss. However, for high pulse frequency, the peak current time happens more frequently, which will lead to the concentration of heat input and inhibit the heat loss, so the molten pool will obtain more continuous heat input and higher temperature. Based on the above mentioned factors, the high temperature under high pulse frequency can be conducive to broadening the molten pool. Hence, the width of deposited wall increases and layer thickness decreases as the pulse frequency increases.

#### 3.1.2. Microstructure

**Figure 4:**
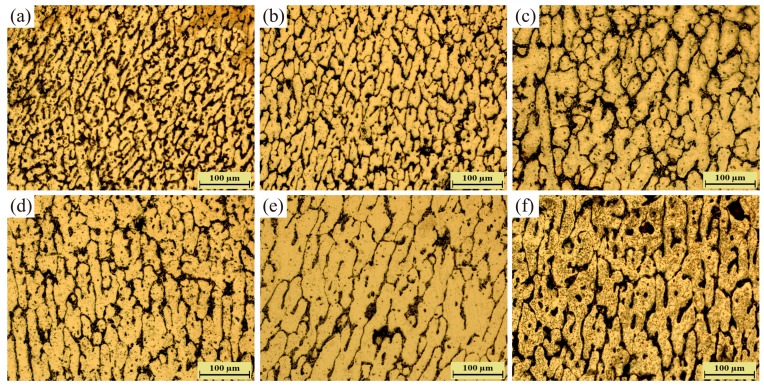
Microstructures of the Al–5Si walls deposited by (**a**) 2 Hz; (**b**) 5 Hz; (**c**) 10 Hz; (**d**) 50 Hz; (**e**) 200 Hz; and (**f**) 500 Hz.

**Figure 5:**
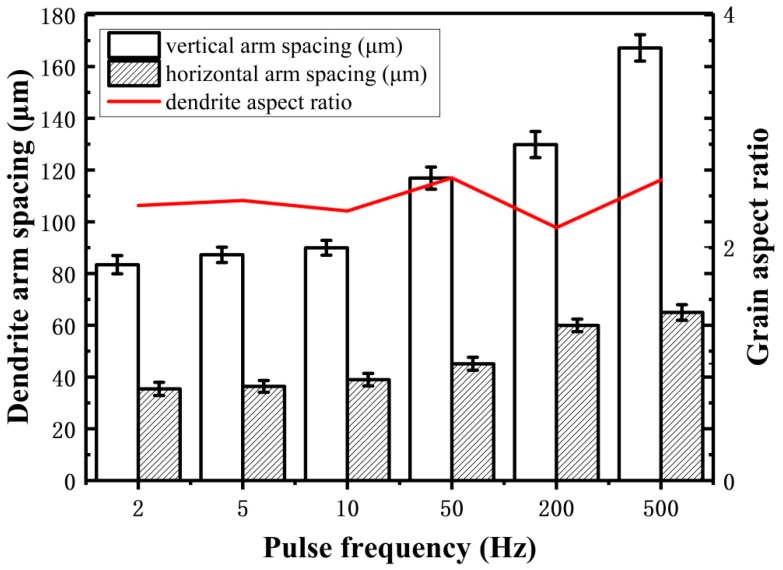
Effect of pulse frequency on the dendrite arm spacing in Al–5Si alloy.

The microstructures of arc additive manufacturing Al–5Si alloy under different pulse frequency are shown in Figure 4. It can be clearly seen that all the grains exhibit dendrites morphology and the grains become coarser with the increase of pulse frequency. The quantitative statistics results of dendrite arm spacing are shown in Figure 5. It shows that the dendrite arm spacing increases with the increase of pulse frequency from 2 Hz to 500 Hz in both directions (horizontal and vertical). Specifically, with the increase of pulse frequency, the horizontal dendrite arm spacing changes from about 30 μm to 60 μm, and the vertical dendrite arm spacing changes from about 80 μm to 170 μm. It is well-known that the grain size is strongly affected by cooling rate, which also determines the tensile properties of the deposited alloy [23]. The heat input is more concentrated at high pulse frequency, which will reduce cooling rate efficiently. So, much more coarse grains can be found at the high pulse frequency. However, low pulse frequency generates the rapid cooling rate in the molten pool. Hence, far more fine grains can be observed at the low pulse frequency.

Besides, it is found that the morphology of dendrites tends to be columnar, and the horizontal arm spacing is much smaller than the vertical arm spacing, as shown in Figure 5. This is because of the high temperature gradient caused by the heat loss through the substrate or previously deposited layers during arc additive manufacturing [24]. The high temperature gradient will promote the formation of columnar grains from the bottom of the molten pool [17]. For the additive manufacturing Al–5Si alloy in this study, the grains exhibit short columnar morphology because the formation of columnar grains is not only associated with the temperature gradient, but also with the alloy composition. Moreover, although the dendrites become coarser with the increase of the pulse frequency, the aspect ratio of dendrites seems to be stable at about 2.2. As known, the dendrites morphology mainly depends on the ratio of temperature gradient to solidification velocity [25]. Hence, the results indicate that the change of pulse frequency has not had significant effect on the ratio of temperature gradient to solidification velocity.

#### 3.1.3. Porosity

**Figure 6:**
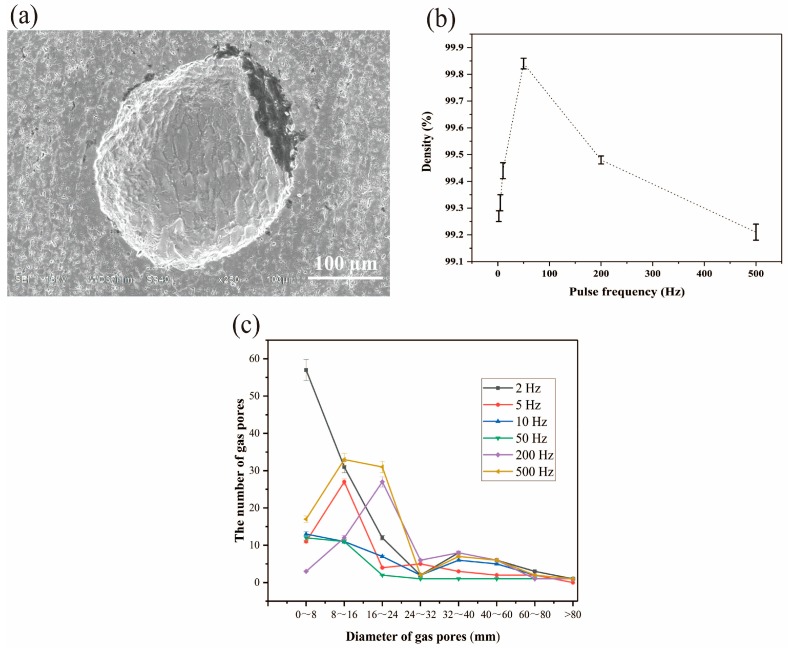
Effect of pulse frequency on porosity: (**a**) Scanning electron microscope (SEM) image shows the gas pores; (**b**) density of the Al–5Si alloy sample; and (**c**) distribution of gas pores with different size.

Figure 6a shows the gas pores in the additive manufacturing Al–5Si alloy samples. The cross section of gas pores are nearly circular. Then, the quantitative analysis of the relationship between the gas pores and pulse frequency is carried out as follows. 

As shown in Figure 6b, the turning point is around 50 Hz. At the low frequency condition (under 50 Hz), the density of samples increases with the increase of the pulse frequency in general. However, at high pulse frequency (above 50 Hz), the density decreases with the increase of pulse frequency. At this pulse frequency, the density is at a maximum (99.85%). Moreover, some relationships between the area of gas pores and the pulse frequency can be found, as shown in Figure 6c. At low frequency, much smaller pores are found in the samples. However, at high pulse frequency, there are more large pores than the small pores. It shows that the pulse frequency can affect the proportion of the size of pores on arc additive manufacturing process.

Apparently, pulse frequency has an appreciable influence on porosity. According to the previous work as reported in the literature [26,27], there are four stages in the formation of gas pores in the manufacturing process, namely, nucleation, growth, detaching, and escaping. Gas pore growth needs to follow the condition that was represented in Equation (1). The detachability mainly depends on infiltration angle *θ*, which is shown in Equation (2). It determines whether the gas pore detaches wholly or not. For the escaping stage, it is recognized as a complex effect of density, curvature radius, and liquid viscosity. The expression of escape speed is shown in Equation (3).

*P_h_* > 1 + 2*σ*_2,g_/*r* (1)

where *P_h_* is the pressure inside of gas pore, *σ*_2,g_ is the surface tension between liquid and gas pore, and *r* is the curvature radius.

cos *θ* = *(σ*_1,g_ − *σ*_1,2_*)*/*σ*_2,g_ (2)

where *θ* is the infiltration angle, *σ*_1,g_ is the surface tension between wall and gas pore, *σ*_2,g_ is the surface tension between liquid and gas, and *σ*_1,2_ is the surface tension between wall and liquid.

*v* = 2 (*ρ*_1_ − *ρ*_2_) *g r*^2^/9*η* (3)

where *v* is the escape speed, *ρ*_1_ is the density of liquid, *ρ*_2_ is the density of gas, *g* is the acceleration of gravity, *r* is the curvature radius of gas pore, and *η* is the liquid viscosity. 

**Figure 7:**
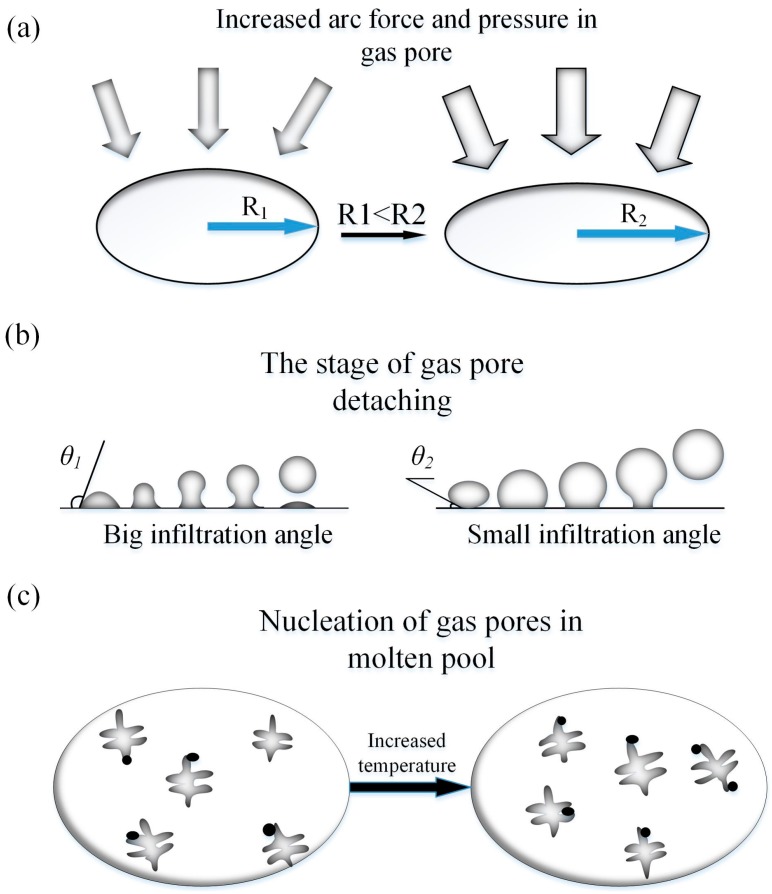
The formation of gas pores with increased pulse frequency: (**a**) Curvature radius; (**b**) infiltration angel; and (**c**) the number of nucleation sites.

This phenomenon can be analyzed as follows:

Firstly, increased pulse frequency can cause the increase of the arc force and arc pressure, so the liquid pressure will increase. Then, the curvature radius of gas pores will be larger, as shown in Figure 7a, surface tension between gas and liquid is constant during the process of gas growth. According to Equation (1), higher pulse frequency will reduce the value of the right side of the inequation. Obviously, higher pulse frequency helps gas pores grow, as reported by Cong B. et al. [15].

Secondly, increased pulse frequency with a larger heat input, which will increase the temperature of molten pool, and the cooling rate will lower simultaneously [28]. We can conclude that high pulse with high temperature will reduce the *σ*_2,g_ (surface tension between liquid and gas pore) and *σ*_1,2_ (surface tension between wall and liquid). Because of the larger curvature, gas pores will gain a larger touching area. As known, larger touching area will increase the *σ*_1,g_. Above all, as shown in Equation (2), with the increase of pulse frequency, *σ*_1,g_ will increase while *σ*_2,g_ and *σ*_1,2_ will decrease. Therefore, high pulse frequency with small infiltration angle will help pores detach, as shown in Figure 7b. According to previous work, liquid viscosity is also affected by temperature. With the temperature increasing, liquid viscosity will decrease. The variation of pulse frequency has no obvious effect on the density of liquid and gas and curvature radius of gas pores increase with the increase of the pulse frequency. It can be seen that with the increase of pulse frequency, the cooling rate will reduce and the escape speed will increase. Gas pores can escape from the molten pool on the condition that escape speed is greater than cooling rate. Nonetheless, the higher the temperature of the molten pool is, the more intense the chemical reacts in molten pool. Besides, higher temperature of the molten pool will increase its capacity of H^+^ absorption, which leads to more nucleation sites of gas pores in the molten pool, as shown in Figure 7c.

Based on the above mentioned reasons, there exists an optimum pulse frequency of porosity minimization. With the increase of pulse frequency (under 50 Hz), it is easier for gas pores primarily to detach and escape from the molten pool. Therefore, density will increase with the increase of pulse frequency (under 50 Hz). For high pulse frequency (above 50 Hz), with the increase of pulse frequency, the gas pores’ capacity to detach will be larger, nonetheless, the number of gas pores’ nucleation sites will increase, more gas pores will form, although the escaping speed is bigger, there is still leaving gas pores. Thus, density decreases with the pulse frequency increasing. At the pulse frequency of 50 Hz, nucleation and escaping of gas pores seem to be more balanced, leading to high-density additive manufacturing aluminum alloys.

#### 3.1.4. Tensile Properties

**Figure 8:**
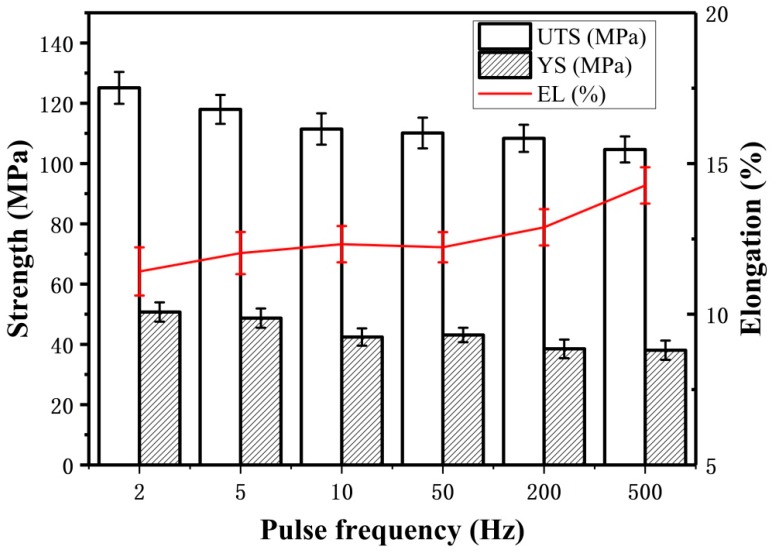
Effect of pulse frequency on tensile properties of arc additive manufacturing Al–5Si alloy. UTS—ultimate tensile strength, YS—yield strength; EL—elongation.

**Figure 9:**
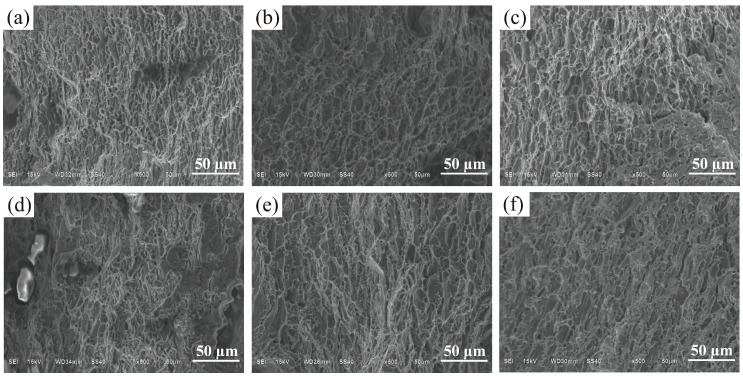
SEM fractographs of tensile samples fabricated with different pulse frequencies: (**a**) 2 Hz; (**b**) 5 Hz; (**c**) 10 Hz; (**d**) 50 Hz; (**e**) 200 Hz; and (**f**) 500 Hz.

From Figure 8, with the pulse frequency decreasing, a good strength was achieved. Concretely speaking, the ultimate tensile strength (UTS) of the samples decreases from 125.1 MPa to 108.6 MPa with an increase in the pulse frequency from 2 Hz to 500 Hz. Meanwhile, the yield strength (YS) of the samples also decreases with an increase in the pulse frequency from 2 Hz to 500 Hz. It is claimed that the finer microstructure will lead to a higher tensile strength [22,29]. When the pulse frequency increases, the grains become coarser, thus the strength decreases. Furthermore, the fracture surface micrographs of the tensile samples at different pulse frequencies are given in Figure 9. It can be seen that far more dimples can be found at low pulse frequency than at high pulse frequency, and the dimples appear deeper at low pulse frequency, which supports the results that the samples fabricated at low frequency have higher strength.

### 3.2. Effect of Arc Current

#### 3.2.1. Macrostructure

Similar to pulse frequency, arc current can also influence the geometry of Al–5Si alloy manufactured by arc additive manufacturing, as shown in Table 4. As arc current increases from 100 A to 175 A, layer thickness decreases from 1.04 mm to 0.79 mm, while the width of samples increases from 5.72 mm to 9.56 mm. This is because with the increase of arc current, molten pool will absorb more heat, which broadens the molten pool and decreases the layer thickness. Besides, the surface morphologies of samples vary only a little under different arc current.

#### 3.2.2. Microstructure

**Figure 10:**
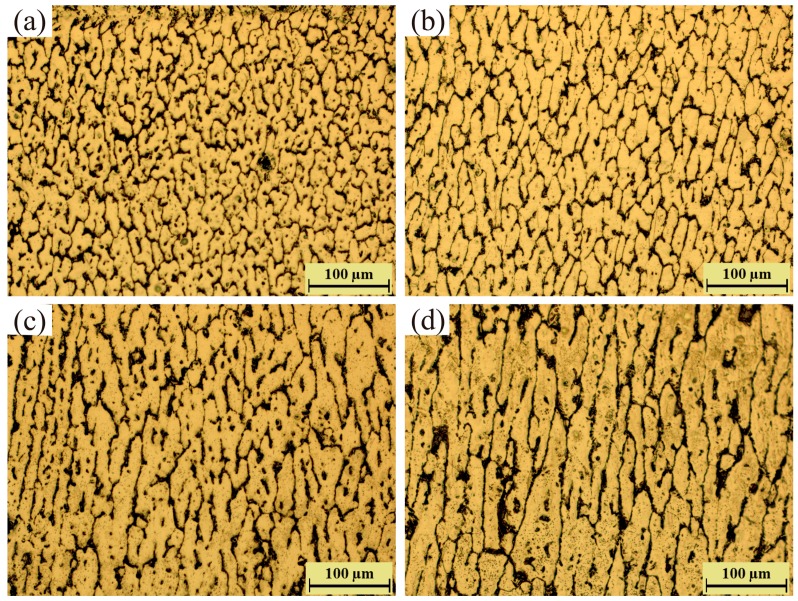
Microstructures of the walls deposited by (**a**) 100 A; (**b**) 125 A; (**c**) 150 A; and (**d**) 175 A.

**Figure 11:**
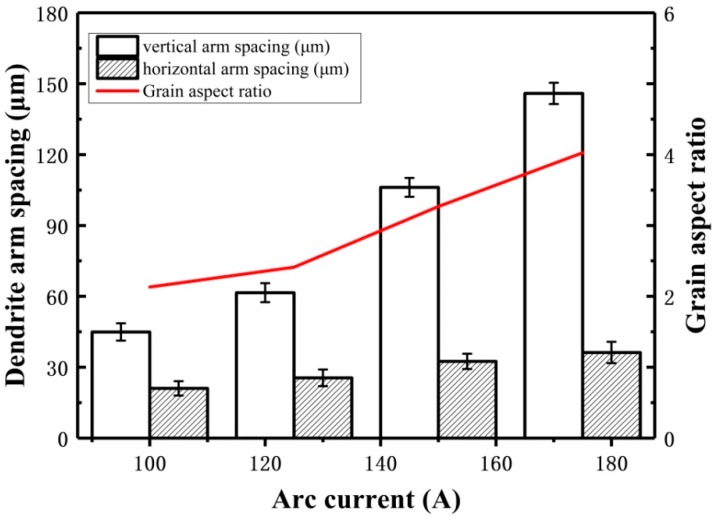
Effect of arc current on the microstructures of deposited layers.

Compared with pulse frequency, arc current can affect grain size and secondary dendrite arm spacing more strongly, as shown in Figure 10. It is obvious that grain size increases with the increase of arc current, as shown in Figure 11. Specifically, both the horizontal arm spacing and vertical arm spacing of grain increase. However, vertical arm spacing appears to have a more clear growth than horizontal arm spacing, which can been seen in Figure 11. Because of the variation of arm spacings in two different directions, grains change from near equiaxed morphology into columnar morphology, as shown in Figure 10.

Obviously, arc current has significant effect on the microstructures of Al–5Si alloys because it controls the heat input to material [30]. Small arc current leads to low heat input, high cooling rate and high nucleation rate, which results in fine grains with near equiaxed morphology. For large arc current, the cooling rate and temperature gradient both decrease, thus the grains exhibit coarse and long columnar morphology.

#### 3.2.3. Tensile Properties

**Figure 12:**
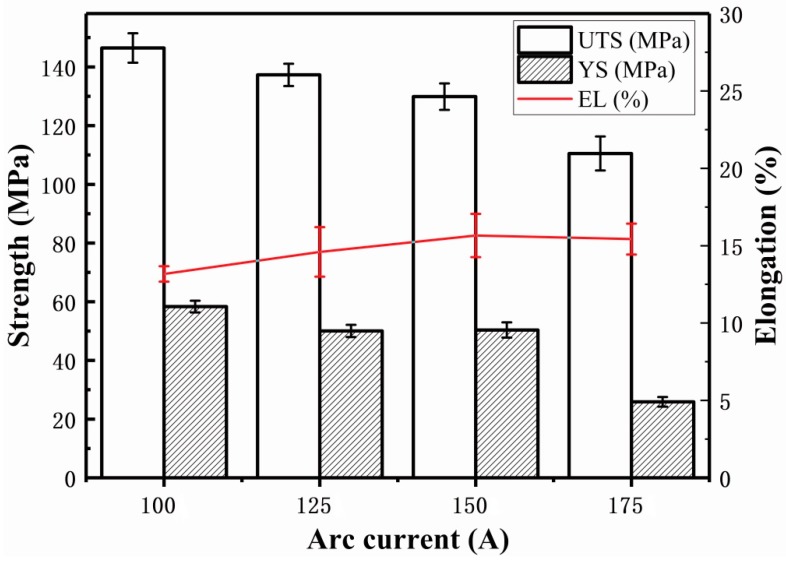
Effect of arc current on tensile properties of deposited of Al–5Si alloy.

**Figure 13:**
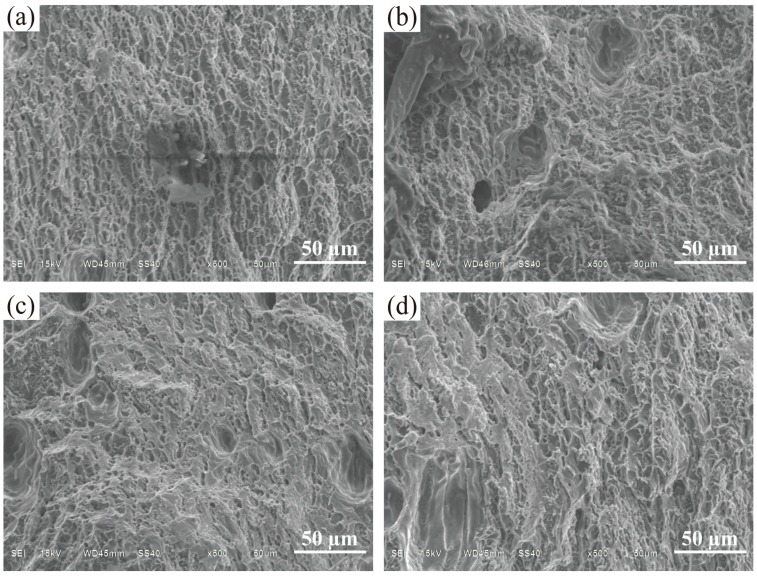
SEM fractographs of tensile samples manufactured with different pulse frequencies: (**a**) 100 A; (**b**) 125 A; (**c**) 150 A; and (**d**) 175 A.

Figure 12 shows that with an increase of the arc current from 100 A to 175 A, the ultimate tensile strength (UTS) of the samples clearly decreases from 146.5 MPa to 110.5 MPa. The yield strength (YS) at failure also decreases in general, but the elongation (EL) increased. Obviously, this is caused by the microstructure changes. According to the Hall–Petch equation [31], the mechanical property of the alloy is directly affected by the average grain size. In other words, the fine microstructures under small arc current leads to high strength but poor tensile ductility [32]. Figure 13 shows the fracture surface micrograph of the tensile samples with different arc currents. It can be seen that far more dimples can be found at low arc current than that at large arc current. Compared with the dimples at large arc current (see Figure 13c,d), the dimples appear deeper at low arc current (Figure 13a,b), which is consistent with the tensile properties.

## 4. Conclusions

In this study, Al–5Si alloy samples were fabricated by arc additive manufacturing with different arc currents and pulse frequencies. The macrostructure, microstructure, porosity, and tensile properties were studied. From this investigation, a few key points can be concluded, as below:

(1) Pulse frequency strongly affects the porosity of arc additive manufacturing Al–5Si alloy. With the increase of pulse frequency, density shows a trend that escalates firstly and attains its maximum in 50 Hz, but declines later because of the relation between pores formation and gas escape.
(2) With the increase of pulse frequency, grains become coarser because of the more centralized heat input, but the grains still exhibit short columnar morphology. Because of the variation of microstructure, tensile strength decreases from 125.1 MPa to 108.6 MPa with the increase of pulse frequency.
(3) Arc current also has a significant impact on microstructure and tensile properties. With the increase of arc current, grains become coarser and change from short columnar morphology to long columnar morphology as a result of more heat input, and hence leads to the decrease of tensile strength from 146.5 MPa to 110.5 MPa.

In summary, coarse grains and gas pores are two main problems that limit the application of arc additive manufacturing Al–Si alloys. Combined with the above results, fine grains and uniform microstructure can be obtained. This paper illustrate that pulse frequency and arc current have a significant impact on the macrostructure, microstructure, porosity, and tensile properties of the samples, which is meaningful for the further application of arc additive manufacturing aluminum alloy.

## Figures and Tables

**Table 1:** Composition of the ER4043 (Al–5Si) filler wire.

Element	Si	Mn	Mg	Cu	Fe	Zn	Al
wt %	5	<0.05	<0.05	<0.05	<0.4	<0.1	Balance

**Table 2:** Deposition parameters for arc additive manufacturing in this study.

Deposition Parameters	Values Ⅰ	ValuesⅡ
Pulse frequency	2, 5, 10, 50, 200, 500 Hz	2 Hz
Peak current	100 A	100, 125, 150, 175 A
Peak time ratio	30%	30%
Base-to-peak current ratio	30%	30%
Wire feed speed	100 cm/min	100 cm/min
Deposition speed	100 mm/min	100 mm/min
Shield gas flow rate	20 L/min	20 L/min

**Table 3:** Effect of pulse frequency on the geometry of deposited layers.

Pulse Frequency (Hz)	Layer Thickness (mm)	Width (mm)
2	0.92 ± 0.02	5.81 ± 0.01
5	0.96 ± 0.03	5.84 ± 0.03
10	0.88 ± 0.02	6.51 ± 0.02
50	0.86 ± 0.03	6.37 ± 0.05
200	0.83 ± 0.04	6.46 ± 0.02
500	0.84 ± 0.05	6.73 ± 0.03

**Table 4:** Effect of arc current on the geometry of deposited layers.

Arc Current (A)	Layer Thickness (mm)	Width (mm)
100	1.04 ± 0.07	5.72 ± 0.02
125	0.83 ± 0.02	5.70 ± 0.01
150	0.80 ± 0.03	8.14 ± 0.07
175	0.79 ± 0.02	9.56 ± 0.03
